# Solitary intraosseous schwannoma of the base and vault of the skull: a summary review of such unusual location

**DOI:** 10.1186/s13569-015-0023-1

**Published:** 2015-02-09

**Authors:** Fadoua Rais, Naoual Benhmidou, Ghizlane Rais, Fadila Kouhen, Khadija Bellahamou, Hasna Loughlimi, Abdelhak Maghous, Sanae Elmejjaoui, Hanan Elkacemi, Tayeb Kebdani, Noureddine Benjaafar

**Affiliations:** Department of Radiotherapy, National Institute of Oncology, Rabat, Morocco; Department of Oncology, National Institute of Oncology, Rabat, Morocco

**Keywords:** Intra-osseous schwannoma, Schwannoma, Skull bone

## Abstract

Intra-osseous schwannoma is a rare mesenchymal tumor. Although, the head and neck region is one of the most common sites for schwannomas, its location at the skull bone is uncommon and accounted for less than 0.2% in the largest series of bone tumors ever reported. Furthermore, it is most often a benign tumor, malignant transformation is exceedingly rare.

Clinical presentation is non-specific, most often symptoms are associated with compression and invasion of adjacent organs. Neuro-imaging features are non-specific and the diagnosis is based on histological examination with immunohistochemical study.

Surgery remains the aim of treatment. However, radiation therapy could be an interesting therapeutic option in unresectable tumors.

This systemic review offers new clinicopathological data useful for better defining the diagnosis and clinicopathological behavior of schwannoma. The purpose of this work is to raise awareness among clinicians adding this clinical entity as a differential diagnosis when a mass of skull bone is identified.

## Introduction

Schwannoma also referred to as neurilmoma, neurinoma, and perineural fibroblastoma is a solitary soft tissue or intra-bone lesion, most often developped from a Peripheral nerve [1].

Schwannoma arising from Schwann cells, first described by Verocay in 1910, is most often a benign tumor, malignant transformation is exceptional [1]. Intraosseous schwannoma is a rare mesenchymal tumor, its location at the skull bone is extremely rare [1-3]. There are few reports of individual cases [1,2,4-6], but no large series from a single institution has been published yet.

## Review

Intraosseous schwannomas are known to account for less than 0.2% of primary bone tumors [1,3]. To the best of our knowledge, only 10 cases without neurofibromatosis disease have been described to date.

Clinical presentation is non-specific, most often symptoms are associated with compression and invasion of adjacent organs. The diagnosis is based on histological examination with immunohistochemical study [1,7]. Surgery is the mainstay of treatment, however, radiotherapy remains the salvage treatment for unresectable tumors [8].

Through this review of the literature, we try to illuminate this extremely rare pathology. It is necessary to distinguish malignant tumors usually occuring in the context of Von Reclinghausen disease, from benign schwannomas [9]. In fact, these entities are different by their clinical and pathological behaviors. In order to avoid confusion between them, we try to treat separately the two entities.

### Classification

The new World Health Organization (WHO) classification [10] of soft tissue tumours, published in 2013, incorporates for the first time peripheral nerve sheath tumours. It was previously included with the 2007 WHO classification of the central nervous system. Overall, the WHO felt that these groups of lesions fitted better under the heading of ‘Soft tissue tumours’, and this allows a more comprehensive classification [11].

According to this classification, schwannoma (including variants) is a begnin entity, whereas malignant schwanoma is included under the name of *malignant peripheral nerve sheath tumour (MPNST)*. This WHO classification of sheath nerve tumours is resumed in Table 1 [12].

**Table 1:** **WHO classification of soft tissue tumours (2013) including for the first time nerve sheath tumours** [12]

**Nerve sheath tumours**	
**Benign**	**Malignant**
Schwannoma (including variants)	Malignant peripheral nerve sheath tumour
Melanotic schwannoma	Epithelioid malignant nerve sheath tumour
Neurofibroma (including variants)	Malignant Triton tumour
Plexiform neurofibroma	Malignant granular cell tumour Ectomesenchymoma
Perineurioma	
Malignant perineurioma	
Granular cell tumour	
Dermal nerve sheath myxoma	
Solitary circumscribed neuroma	
Ectopic meningioma	
Nasal glial heterotopia	
Benign Triton tumour	
Hybrid nerve sheath tumours

1. Benign Schwannoma.

### Patient demographics

Intraosseous schwannomas affect males and females equally, with patient age ranging from 2 to 65 years. The mandible is the most common location, followed by sacrum. Other sites such as vertebra, ulna, humerus, femur, tibia, patella, scapula, rib, and small bones of the hands have been reported [13].

Approximately 25% of the reported cases originate from the head and neck region [1]. However, intra-osseous schwannoma of the skull bone are very rare, accounted for less than 0.2% of primary bone tumors [1-3]. Data are available from published isolated case reports and small case series. The first documented case in the literature was published by Solodnik and al in 1986 [5,14].

In one of the most important reviews of intraosseous schwannoma, Gordon and al presumed that the diagnosis of this entity might be missed because of its rarity and atypical roentgenologic appearance [15].

### Histogenesis

Schwannoma is associated with sensory nerves. Therefore, the exceedingly rare occurrence of intraosseous schwannoma is due to the low density of sensory nerve fibers within bone [2,13,16]. Schwannomas can involve bone by 3 mechanisms [1,2,16].

- Tumor may arise centrally within the bone,
- Tumor can arise within the nutrient canal and produce canal expansion: tumors grow in a dumbbell-shaped configuration, enlarging the canal
- Extraosseous tumors can cause secondary erosion of bone: a soft tissue or periosteal tumor may cause secondary erosion of the bone and penetration into.

However, the histological origin of such intra-osseous schwannoma remains controversial. In fact, hyperplastic schwann cells of the perivascular nerve plexus, especially of autonomic nerves could explain the development of meningeal schwannoma [17], and even intraosseous schwannoma [18]. Also, schwannosis which is an hamartomatous lesion consisting of Schwann cells and related reticulin fibers could not be excluded in histogenesis of such lesion [5,19].

### Histopathology

Histologic features of intraosseous schwannomas are usually similar to that observed for soft-tissue schwannomas including Antoni A type (characterized by Schwann cells closely packed and arranged in bundles or rows with palisading nuclei) and Antoni B type (with less number of cells and disorganization of fusiform cells dispersed in a loose and random fashion) tissue arrangements [1,13].

Schwannoma is an encapsulated neoplasm, characterized by slow growing and low invasive potential, it rarely undergoes malignant transformation [20,21].

Important features to suggest a benign diagnosis include absence of infiltrative margins (sharp circumscription), perivascular hyalinization, disproportionally high cellularity when compared to the degree of nuclear atypia or mitotic activity, and strong and uniform immunostaining for S-100 protein [20,22].

A diagnosis of schwannoma with uncertain malignant potential could be evoked in front of a locally aggressive growth pattern in a histologically benign-appearing tumor [20].

### Immunohistochemistry

The histological diagnosis of schwannoma is difficult because of the similarity with other sarcomas, so pathologists use S100 protein which is specific for nervous cells [7,23].

In fact, Immunohistochemically, schwannoma is nearly uniformly positive for the S-100 protein and for vimentine [1].

### Clinical features

Clinical symptoms of schwannoma are not specific and depend on the location. They are most often related to compression of adjacent organs or of their invasion [7].

The skull bone is an exceedingly rare site for intraosseous schwannoma. Only 10 cases of this location have been reported previously (Table 2).

**Table 2:** **Solitary intraosseous schwannoma of the vault and the base of the skull patient characteristics**

**Authors**	**Age**	**Sex**	**Site**	**Principals symptoms**	**Treatment**	**Follow up**	**Outcome**
**Solodnik et al. (1986)** **[** 14 **]**	59-year	Male	Petrous apex	Headache, tinnitus, and unsteadiness.	Sub-total excision	?	Good
**Schiffer et al. (1991)** **[** 6 **]**	3-year	Male	Fronto-orbital bone	A non tender mass	Total good excision	1 year	Good
**Unni KK (1996)** **[** 26 **]**	Report of two cases	?	Skull bone	?	?	?	?
**Celli et al. (1998)** **[** 24 **]**	14-year	Male	Frontal bone	Hard non tender mass	Complete excision	2 year	Good
	3-year	Male	Occipital bone	Hard non tender mass	Complete excision	14 year	Good
**Ersahin et al. (2000)** **[** 13 **]**	4-year	Male	Parietal bone	A non tender solid mass	Totalexcision	2 year	Good
**El bahy and al (2004)** **[** 5 **]**	40-year	Male	Spheno-orbital bone	Proptosis, swelling of the frontozygomatic junction	Complete excision	?	Good
**Goyal R et al. (2008)** **[** 2 **]**	11-year	Male	Frontal bone	Forehead swelling	Complete excision	6 month	Good
**Amita et al. (2014)** **[** 25 **]**	41-year	Female	Frontal bone	**Paresthesia of limbs, hemicranial headache, generalized tonic-clonic seizures**	Complete excision	3 months	Good

The first documented case in the literature was published by Solodnik and al in 1986, they described an intraosseous neurinoma of petrous apex of a 59 year-old man with headache, tinnitus in the right ear, and unsteadiness [14].

Ersahin and al reported a case of intraoessous schwannoma of parietal bone of a 4-year-old boy presented with a lump on the right side of his head with a mass witch enlarged in size progressively [13].

The patient reported by Schiffer and al in 1991 was a 3-year-old boy with a non tender mass in the left fronto-orbital region [6].

Sphenoidal schwannoma resembles other primary tumors of the sphenoid in symptomatology and physical signs [5,6]. In fact, El bahy and al reported a case of spheno-orbital bone of a 40-year-old man with proptosis and impairment of lateral gaze movement of his left eye and a palpable soft non tender swelling at the left frontozygomatic junction [5].

Two others cases was reported by Celli.P and al, the first of occipital bone and the second of frontal bone, both of them presented with a hard non tender mass [24].

Another case of frontal bone reported by Goyal R and al of an 11-year-old boy presented with forehead swelling that increased progressively during a period of 2 years [2].

Recently R.Amita published another case of frontal bone in a 41-year-old female presented with insidious onset intermittent paresthesia in right upper and lower limbs and left hemicranial headache with two episodes of generalized tonic-clonic seizures [25]. Unni and al reported 2 cases of intraosseous schwannoma of the skull but without more detailed information [26].

Physical examination, in all reported cases was normal, especially without neurological deficits.

Approximately 10% of schwannomas are associated with multisystem disorders such as neurofibromatosis, schwannomatosis, multiple meningiomas, and Carney complex [20]. However, these disorders have not been present in anyone of previously cited cases.

### Neuro-imaging features

The preoperative diagnosis is often difficult, as the neuro-imaging features are non-specific, but it contributes to evaluate the extent and determine infiltration of the adjacent structures [1].

At CT scan, intraosseous schwannomas are most often present as a lytic lesion, with sclerotic borders, or as a sharply demarcated defect that often expand the involved bone. Perilesional sclerosis may surround the area of central lucency [2], and sometimes spotted calcifications may be seen [13]. Furthermore, it may be presented as a soft-tissue mass with bone erosion [13].

MRI showed an inhomogeneous lesion on T1-weighted spin-echo images, hyperintense signal on T2-weighted spin-echo images, and isointense on T2-fast images. The lesion showed peripheral intense enhancement with gadolinium [13,24].

2. Malignant schwannoma or malignant peripheral nerve sheath tumour (MPNST).

Malignant peripheral nerve sheath tumour (MPNST) is a rare aggressive tumor, which represents 5% of soft tissue sarcomas. Nevertheless, there was a resurgence of this condition in patients with Von Recklinghausen disease [7].

MPNST of the head and neck is extremely rare [27] and has not been reported in an intraosseous skull bone location. Although occasional neoplasms have been reported in the maxilla, mandible, palate and other sites. The mandible is the most commonly invaded structure in the oral area [27-30].

Otherwise, solitary malignant schwannoma is very uncommon. In a large series of 232 patients with solitary malignant schwannomas, not associated with Von Recklinghausen’s disease, reported by Tapas and all [31], eighteen (7%) tumors were in the head and neck but none of them was intraosseous.

The microscopic diagnosis of MPNST is difficult because the tumors often consist of fusiform elements packed in interlacing bundles and resemble fibrosarcoma [31].

Malignancy is retained on cell pleomorphism, high number of mitosis and capsular infiltration [7,23]. Also, the lack of well-delimited borders and encapsulation, invasion signs, significant atypia and necrosis suggest malignancy [32]. Large areas of necrosis and high mitotic activity are typical [21].

In contrast to benign and locally aggressive schwannomas, which demonstrate uniform immunoreactivity for S-100 protein, malignant entity may show patchy or absent immunoreactivity for this antigen [10].

There is no clinical findings characteristic of MPNST. In head and neck reported cases, the chief reason the patient seeks advice is a soft tissue mass which is gradually enlarging [31].

Prognostic factors are tumor size, grade of malignancy, speed of clinical evolution, degree of cellular pleomorphism and mitotic activity [7].

For malignant peripheral nerve sheath tumor (MPNST), the 5-year survival is ranged from 50 to 55% in patients without neurofibromatosis but it is reduced to less than 20% in patients with this disease [7,33]. They tend to recur (40%–65%) and metastasize (40%–80%) [34].

### Treatment

All reported cases in the literature were benign, therefore intraosseous schwannoma was treated by complete excision in most cases. They registered a good outcome with no local recurrence within a follow-up that ranged from 3 months to 14 years.

The optimal treatment for intraosseous schwannoma has not been well established given the rarity of this entity. Total resection of intraosseous schwannomawas described as a sufficient treatment, in particular for limited tumors without invasion of adjacent structures. However, only volume reduction, without total removal or tumor control can be achieved with a surgical approach in others cases [35].

Radical excision is based on location, shape, size and local spread. The tumor plus its bed and any attached structures should be resected en bloc [31].

As alternative treatment, stereotactic radiosurgery and radiotherapy may be performed, when the tumor is unresectable. In fact, the extent of the resection and potential morbidity must be considered in addition to accessing the region of interest [35].

Therefore, radio surgery may be suggested when microsurgical resection is contraindicated, as well as for patients with serious comorbidities or underlying neurofibromatosis, which often involves multiple lesions [4].

The effect of radiotherapy has been studied in various schwannomas and has been shown to be effective in preventing tumor growth, although long-term assessments of the benefits in patient quality of life remain uncertain. Little is known about the comparative efficacy of stereotactic radiotherapy to surgery [8].

The use of pre or post-operative adjuvant therapy is not yet well codified, but in case of incomplete resection, radiotherapy can be envisaged [1,7].

The role of chemotherapy in advanced malignant peripheral nerve sheath tumor (MPNST) is unclear. Most MPNSTs are biologically high-grade sarcomas that tend to recur (40%–65%) and metastasize (40%–80%). MPNST usually metastasize most commonly to the lungs [34].

Because of the rarity of MPNST, consistent data regarding chemotherapy sensitivity are lacking. As for other unresectable and metastatic soft tissue sarcoma, doxorubicin and ifosfamide are generally considered to be the most active chemotherapeutic agents.

In a recent study of EORTC soft tissue and bone sarcoma group study, that compares the outcome of patients with advanced MPNST, with those with other soft tissue sarcoma histological subtypes, the findings showed that, compared with standard first-line doxorubicin, the doxorubicin–ifosfamide regimen had the best response, whereas ifosfamide had the worst prognosis. Results of an ongoing NCI multicenter phase II trial (NCT00304083) that studies combination chemotherapy with doxorubicin, etoposide and ifosfamide in unresectable (stages III–IV) adult MPNST are awaited [34].

## Conclusion

The purpose of this review is to offer new clinicopathological data of intraosseous schwannomas of skull bone, useful for better defining the diagnosis and biological behavior of schwannoma specially in such unusual location.

Surgery remains the main treatment. External beam radiation could be an interesting therapeutic option for unresectable tumors. This paper aims to raise awareness among clinicians adding this clinical entity as a differential diagnosis when a mass of skull bone is identified.

## Abbreviations

MPNST: malignant peripheral nerve sheath tumor
WHO: World Health Organization
EORTC: European Organization for Research and Treatment of cancer
